# Downstream Alternate Start Site Allows N-Terminal Nonsense Variants to Escape NMD and Results in Functional Recovery by Readthrough and Modulator Combination

**DOI:** 10.3390/jpm12091448

**Published:** 2022-09-01

**Authors:** Alyssa Bowling, Alice Eastman, Christian Merlo, Gabrielle Lin, Natalie West, Shivani Patel, Garry Cutting, Neeraj Sharma

**Affiliations:** 1 McKusick-Nathans Department of Genetic Medicine, Johns Hopkins University School of Medicine, Baltimore, MD 21205, USA; 2 Division of Pulmonary and Critical Care Medicine, Department of Medicine, Johns Hopkins Hospital, Baltimore, MD 21205, USA

**Keywords:** alternate start, NMD, readthrough, CFTR correctors, CFTR potentiators, transcript stability

## Abstract

Genetic variants that introduce premature termination codons (PTCs) have remained difficult to therapeutically target due to lack of protein product. Nonsense mediated mRNA decay (NMD) targets PTC-bearing transcripts to reduce the potentially damaging effects of truncated proteins. Readthrough compounds have been tested on PTC-generating variants in attempt to permit translation through a premature stop. However, readthrough compounds have not proved efficacious in a clinical setting due to lack of stable mRNA. Here, we investigate N-terminal variants in the cystic fibrosis transmembrane conductance regulator (*CFTR*) gene, which have been shown to escape NMD, potentially through a mechanism of alternative translation initiation at downstream AUG codons. We hypothesized that N-terminal variants in *CFTR* that evade NMD will produce stable transcript, allowing CFTR function to be restored by a combination of readthrough and protein modulator therapy. We investigate this using two cell line models expressing *CFTR*-expression minigenes (EMG; HEK293s and CFBEs) and primary human nasal epithelial (NE) cells, and we test readthrough compounds G418 and ELX-02 in combination with CFTR protein modulators. HEK293 cells expressing the variants E60X and L88X generate CFTR-specific core glycosylated products that are consistent with downstream translation initiation. Mutation of downstream methionines at codons 150 and 152 does not result in changes in CFTR protein processing in cells expressing L88X-*CFTR*-EMG. However, mutation of methionine at 265 results in loss of detectable CFTR protein in cells expressing E60X, L88X, and Y122X *CFTR*-EMGs, indicating that downstream translation initiation is occurring at the AUG codon at position M265. In HEK293 stable cells harboring L88X, treatment with readthrough compounds alone allows for formation of full-length, but misfolded CFTR protein. Upon addition of protein modulators in combination with readthrough, we observe formation of mature, complex-glycosylated CFTR. In CFBE and NE cells, addition of readthrough ELX-02 and modulator therapy results in substantial recovery of CFTR function. Our work indicates that N-terminal variants generate stable *CFTR* transcript due to translation initiation at a downstream AUG codon. Thus, individuals with CF bearing 5′ nonsense variants that evade NMD are ideal candidates for treatment with clinically safe readthrough compounds and modulator therapy.

## 1. Introduction

Clinical approval of Trikafta (elexacaftor-tezacaftor-ivacaftor) has revolutionized treatment for a large portion (90%) of individuals with cystic fibrosis (CF) and drastically improved outcomes [1]. Elexacaftor and tezacaftor are correctors which facilitate folding of the cystic fibrosis transmembrane conductance regulator (CFTR) and assist trafficking of mature CFTR protein to the cell surface. Ivacaftor, a potentiator, increases the probability of open channel conformation to increase chloride ion conductance. These modulator therapies rely on the presence of targetable CFTR protein. However, many individuals carry one or two variants that are not predicted to produce full-length protein due to the presence of a premature termination codon (PTC). Nearly half of disease-causing variants in *CFTR* are predicted to introduce a PTC. This mechanism of disease is not unique to CF, and PTCs are associated with many inherited and acquired severe disease phenotypes, including Duchenne muscular dystrophy (DMD) and β-thalassemia [2,3,4].

PTCs cause the ribosome to stop translation prematurely, often resulting in production of a non-functional, truncated protein, which can have dominant negative effects [5,6,7,8]. Nonsense mediated mRNA decay (NMD) targets mRNA bearing PTCs for degradation, thereby preventing these potentially damaging effects [9,10]. However, some PTC-bearing transcripts could produce functional protein if degradation does not occur, and in the case of CF, especially if this protein product can be targeted by CFTR modulators.

Small molecules drugs can be used to promote ‘read through’ of PTCs by allowing insertion of a near-cognate tRNA and blocking termination factors [11,12]. This causes translation to continue and results in formation of a full-length protein product. One class of these readthrough drugs are aminoglycosides. Aminoglycosides are used as antibiotics, as they interfere with bacterial ribosomes by blocking translation initiation and promoting miscoding [13,14]. However, some aminoglycosides can also promote miscoding within eukaryotic systems [15]. For this reason, select aminoglycosides have been targeted for use as readthrough therapeutics. However, they have remained limited as a treatment option for PTC-causing variants due to several limitations. Surrounding sequence context, RNA structures, and RNA modifications around a PTC greatly influence the effectiveness of readthrough at the stop codon, and also impact the likelihood of readthrough at a normal termination codon [16,17,18,19,20,21,22,23,24]. Additionally, sustained higher doses of aminoglycosides that are necessary for effective readthrough have been shown to induce both nephron- and ototoxicity [25,26]. Other classes of readthrough molecules have been tested for efficacy, including the oxadiazole compound PTC124. PTC124 was found to be safe for therapeutic use and has minimal side-effects [27,28]. However, PTC124 has not shown significant clinical improvement for both CF and DMD [11,29]. ELX-02 is a eukaryotic ribosomal selective glycoside that is currently in phase 2 clinical trials in individuals with CF carrying one or two nonsense variants [30]. Preliminary studies show increased readthrough activity and reduced toxicity compared to G418 due to the specificity of ELX-02 to cytoplasmic ribosomes as opposed to mitochondrial ribosomes, which is believed to be the key determinant of toxicity of aminoglycosides [31]. However, additional studies need to be completed to determine efficacy of ELX-02 as a therapeutic option. Overall, limitations with readthrough molecules have hindered their advancement as a viable treatment option. In addition to these limitations, there must be available transcript for readthrough molecules to act on and for translation to occur, but NMD limits the availability of mRNA. Thus, efforts should be focused on targeting *CFTR* variants that evade NMD, as readthrough therapeutics could be an effective treatment option.

There are a cluster of variants within the 5′ region of *CFTR* that are able to naturally escape NMD [32]. Since translation can initiate downstream at alternative start codons [33,34,35,36], we first tested whether transcripts bearing these variants resulted in synthesis of an N-terminally truncated protein that might be activated by CFTR modulators. We next considered whether these nonsense variants are responsive to readthrough since they do not induce NMD, and folding defects created by introduction of an alternate amino acid at the PTC might be resolved by modulator therapy. We explored readthrough potential in variants that evade NMD using two cell line models expressing *CFTR*-expression minigenes (EMGs) and primary nasal epithelial (NE) cells using readthrough compounds G418 and ELX-02. We show that stable mRNA facilitates effective readthrough and triple combination therapy augments function of CFTR readthrough products to therapeutic levels.

## 2. Materials and Methods

### 2.1. Expression Minigene Creation

Expression minigene plasmids were created as previously described [32]. *CFTR*-EMGi1-i5 contained abridged intron 1 (216 bp of 5′ and 212 bp of 3′), abridged intron 2 (311 bp of 5′ and 264 bp of 3′), abridged intron 3 (374 bp of 5′ and 456 bp of 3′), abridged intron 4 (307 bp of 5′ and 333 bp of 3′), and full-length intron 5 (882 bp). E60X, L88X, and Y122X variants were individually introduced to EMG plasmids through site-directed mutagenesis (SDM) as previously described [32]. Mutagenic primers were designed using QuickChange Primer Design tool. Primers were used to PCR-amplify EMG plasmid, followed by digest with *DpnI*, transformation of XL10-Gold ultracompetent cells (Agilent, Cedar Creek, TX, USA), and selection of colonies on LB-Ampicillin plates (Quality Biologicals, Gaithersburg, MD, USA). DNA minipreps were prepared (Denville Spinsmart Plasmid Miniprep DNA Purification Kit, Swedesboro, NJ, USA) and presence of the variant of interest was confirmed by Sanger sequencing. Selected miniprepped plasmid was used to transform XL10-Gold ultracompetent cells, and DNA maxipreps were prepared (Qiagen Plasmid Plus Maxi Kit, Hilden, Germany). Sanger sequencing was used to verify sequence of entire plasmid and confirm presence of the variant of interest.

### 2.2. Transient Transfection of HEK293 Cells

Plasmids were transiently transected into HEK293 cells using Lipofectamine 2000 (ThermoFisher Scientific, Carlsbad, CA, USA). Protein lysates were collected 48 h after transient transfection.

### 2.3. HEK293 Stable Cell Line Creation

Plasmids were stably transfected into Human Embryonic Kidney (HEK293) cells containing a Flp Recombinase Target (FRT) integration site to allow for site-specific recombination. HEK293 parental cells do not endogenously express *CFTR*. Transfection was performed using Lipofectamine 2000 (ThermoFisher Scientific, Carlsbad, CA, USA). Cells with integrated *CFTR*-EMG were selected for using media containing Hygromycin B, and resistant colonies of cells were isolated and propagated into individual cell lines. Integration of full-length *CFTR*-EMG was confirmed by PCR amplification of genomic DNA, and subsequent protein studies were performed.

### 2.4. Immunoblotting

Total protein was collected from cells using cOmplete Lysis-M mammalian protein extraction reagent containing protease inhibitor cocktail tablet (Millipore Sigma, Mannheim, Germany). Protein concentrations were estimated using the Microplate BCA Protein Assay kit (ThermoFisher Scientific, Rockford, IL, USA). Immunoblotting was performed by denaturing 40 μg of protein at 37 °C for 30 min in Laemmli sample buffer (Bio-Rad, Hercules, CA, USA) containing 200 mM DTT. Samples were run on a 7.5% Criterion TGX precast protein gel (Bio-Rad, Hercules, CA, USA) at 140 V for ~2 h. Protein was transferred to PVDF membrane (Bio-Rad, Hercules, CA, USA) with the Trans-Blot Turbo Transfer System (Bio-Rad, Hercules, CA, USA). The membrane was blocked overnight in 5% nonfat dry milk, PBS and 1% Tween-20 (PBS-T). Membranes were then probed with either mouse monoclonal anti-CFTR antibody 596 (UNC, Chapel Hill, NC, USA) diluted to 1:5000 or rabbit monoclonal anti-sodium/potassium-ATPase (Abcam, Cambridge, UK) diluted to 1:50,000. Membranes were then exposed either anti-mouse secondary antibody (Cytiva, Amersham, UK) diluted to 1:150,000 or antirabbit secondary antibody (Cytiva, Amersham, UK) diluted to 1:100,000. Membranes were imaged on chemiluminescence film using ECL Primer Western Blotting Detection Reagent (Cytiva, Amersham, UK).

### 2.5. CFBE Stable Cell Creation

CF bronchial epithelial (CFBE41o-) cells containing an FRT integration site were used to create stable cell lines expressing *CFTR*-EMG as previously described [32,37,38,39,40]. In brief, CFBE41o- were maintained in complete media with 100 µg/mL Zeocin. Cells were co-transfected with *CFTR*-EMG plasmid and pOG44 (Flp-recombinase plasmid; ThermoFisher Scientific, Carlsbad, CA, USA) using Lipofectamine LTX (ThermoFisher Scientific, Carlsbad, CA, USA). Cells were split 1:4 after 48 h and introduced to media containing Hygromycin B after 72 h. Cells with Hygromycin B resistance formed individual clones which were isolated and propagated into individual cell lines. RNA was extracted from each cell line and reverse transcribed into cDNA. PCR was performed on cDNA using overlapping primer sets spanning *CFTR* to confirm full-length *CFTR* integration. PCR was also performed to confirm disruption of the FRT site. Sanger sequencing was performed on PCR fragments containing the desired variant to confirm presence of that variant of interest, followed by subsequent RNA and protein studies.

### 2.6. CFTR Functional Assessment in CFBE Stable Cells

CFTR function was measured by short circuit current measurements in CFBE stable cell lines as previously described [38,39]. Cells were plated onto Snapwell filters and fed daily on both apical and basolateral sides until transepithelial resistance reached ~200 μΩ at approximately 5–6 days. Cell were treated with following reagents: (1) Readthrough agent (G418 at varying doses—3, 6, 12, 25, 50, 100, 125, 200, 250, 400, 500, 750, and 1000 µM or ELX-02 at varying doses—25, 50, 100 and 200 µM), (2) CFTR correctors (elexacaftor (elexa) and tezacaftor (teza), 3 µM each), and (3) CFTR potentiator (ivacaftor (iva), 10 µM). Cells were treated with readthrough agents (G418 and ELX-02) and CFTR correctors (elexa and teza) either alone or in combination for 24 h. Ivacaftor treatment was given at the time of functional measurement. Readthrough agents are water soluble, while CFTR modulators are DMSO soluble; thus media alone (untreated) or DMSO (0.06%) were used as vehicle controls. It is noted that combination of elexacaftor, tezacaftor and ivacaftor marketed as Trikafta, is a triple combination therapy for individuals with CF who harbor F508del-gating and -residual function genotypes [41,42]. Snapwell filters were mounted into Ussing chambers (Physiological Instruments, Reno, NV, USA) and short circuit currents were measured with a VCC MC6 or VCC MC8 mutlichannel voltage-current clamp amplifier (Physiologic Instruments, Reno, NV, USA). A chloride gradient was created by adding a low chloride solution to the apical chamber and a high chloride solution to the basolateral chamber. Buffers were maintained at 37 °C and air was bubbled to introduce circulation. After currents equilibrated, 10 μM forskolin (Selleckchem, Houston, TX, USA) was added to the basolateral chamber to stimulate production of cAMP, resulting in activation of CFTR channels. After currents plateaued, 10 μM Inh-172 (Selleckchem, Houston, TX, USA) was added to the apical chamber to block CFTR-mediated current. Acquire and Analyze software (Physiologic Instruments, Reno, NY, USA) was used to acquire data, and drop in current (ΔI_sc_) after addition of Inh-172 was used to quantify CFTR-specific function.

### 2.7. Primary Nasal Epithelial Cell Collection

Primary nasal epithelial cells were collected from individuals with CF following IRB protocols at Johns Hopkins University, Baltimore (IRB# 00116966). An experienced physician performed endoscopic procedures to harvest NE cells from individuals after informed consent was obtained. NE cells were collected from the mid-part of the inferior turbinate of both nostrils by brushing with interdental brushes, after spraying a topical anesthetic on the nasal mucosa.

### 2.8. Isolation, Expansion, and Culture of Primary Human Nasal Epithelial Cells

Primary human NE cells were harvested from individuals with CF. Nasal epithelia were conditionally reprogrammed as previously described [32,43,44,45]. Brushes were washed with propagation media and cells were dislodged from the brush. Cells and media were collected into a conical tube and cells were pelleted by centrifugation. Brushes were placed in a conical tube with PBS and vortexed to collect additional cells from the brush. This PBS was used to resuspend the pelleted cells, and cells were pelleted again by centrifugation. The pellet was resuspended with 5 mL Accutase and placed in a 37 °C water bath for five minutes. Accutase was inactivated by the addition of propagation media and cells were pelleted by centrifugation. Cells were resuspended in propagation media containing Rho-associated kinase (ROCK) inhibitor (Y-27632) and were plated into a flask containing irradiated 3T3 mouse fibroblast feeder cells. Feeder layer cells were previously irradiated in the presence of 30 Gy (CIXD Biological Irradiator, Bio-Rad, Singapore; 220 V, 13 A, 468 s). This combination of reagent Y and irradiated mouse fibroblast cells resulted in conditional reprogramming of the primary human NE cells to allow for their propagation in culture for multiple passages. NE cells were then seeded onto snapwell filters (Costar, Kennebunk, ME, USA; #3801) coated with collagen type IV (~300,000 cells/filter). Upon confluency (5–7 days), media was changed from propagation media to PneumaCult-ALI-S media (StemCell, Vancouver, BC, Canada; #05050). To establish air-liquid interface (ALI) culture, media was removed from the apical side and apical surface was washed with PBS. Basolateral media was changed every 48 h. Cells were maintained at 37 °C and 5% CO_2_ and grown on ALI culture for 21–28 days to reach complete differentiation.

### 2.9. Immunostaining and Confocal Microscopy

Primary NE cells growing on air-liquid interface culture for 21–28 days were fixed with 4% paraformaldehyde for 20 min, washed extensively with PBS and permeabilized with 0.25% Triton X-100 for immunostaining. Nonspecific binding sites were blocked with 2.5% normal goat serum (Invitrogen, Waltham, MA, USA; #16201). Cells were stained with primary antibodies against mouse- acetylated-α-Tubulin (Abcam, Cambridge, UK; ab24610) and rabbit-ZO1 (Life Technologies, Rockford, IL, USA; #402200), washed with PBS, and thereafter incubated for 30 min with Alexa Fluor 488 anti-mouse (Invitrogen, Waltham, MA, USA; #A10684) and Alexa Fluor 594 anti-rabbit (Invitrogen, Waltham, MA, USA; #A11012) secondary antibodies respectively. Filters were mounted with Prolong Gold antifade reagent with DAPI (Invitrogen, Waltham, MA, USA; #P36931). The specimens were visualized on a Zeiss 510 confocal microscope (Zeiss, Thornwood, NY, USA; microscope located at the Johns Hopkins School of Medicine Microscope Facility) using 63× magnification.

### 2.10. Scanning Electron Microscopy

Primary human NE cells growing on air-liquid interface culture for 21–28 days were fixed with 2.5% glutaraldehyde, 100 mM sod cacodylate, and 3 mM MgCl_2_. SEM images were visualized at Johns Hopkins School of Medicine Microscope Facility.

### 2.11. RNA Isolation

Primary human NE cells growing on air-liquid interface culture for 21–28 days were lysed by addition of TRIzol (ThermoFisher Scientific, Carlsbad, CA, USA). Chloroform was added and centrifugation separated aqueous and organic phases. The aqueous phase containing RNA was collected and mixed with ethanol. The mixture was transferred to Spinsmart RNA binding columns, and RNA was further purified following manufacturer’s instructions (Macherey-Nagel, Duren, Germany). RNA content and purity were assessed using Nanodrop (ThermoFisher Scientific, Madison, WI, USA).

### 2.12. CFTR Transcript Analysis on Primary Nasal Cells

Complementary DNA (cDNA) was prepared from 500 ng total RNA using iScript cDNA synthesis kit, following manufacturer’s instructions (Bio-Rad, Hercules, CA, USA). Reverse transcription polymerase chain reaction (RT-PCR) was performed using primers that amplify the region surrounding L88X (*CFTR* exons 1–4). Sanger sequencing was performed on an ABI Prism 3100 Genetic Analyzer using the ABI PRISM BigDye Terminator Cycle Sequencing kit (ThermoFisher Scientific, Waltham, MA, USA). Relative *CFTR* transcript abundance was evaluated in a qualitative manner.

### 2.13. CFTR Functional Assessment in Primary Nasal Cells

Well-differentiated human NE cells on ALI culture (21–28 days) were treated with following reagents: (1) Readthrough agent (G418, 100 µM and 400 µM or ELX-02, 100 µM and 200 µM), (2) CFTR correctors (elexacaftor (elexa) and tezacaftor (teza), 3 µM each), and (3) CFTR potentiator (ivacaftor (iva), 10 µM). Cells were treated with readthrough agents (G418 and ELX-02) and CFTR correctors (elexacaftor and tezacaftor) either alone or in combination for 24 h. Ivacaftor treatment was given at the time of functional measurement. Readthrough agents are water soluble, while CFTR modulators are DMSO soluble; thus media alone (untreated) or DMSO (0.06%) were used as vehicle controls. It is noted that combination of elexacaftor, tezacaftor and ivacaftor marketed as Trikafta, is a triple therapy for individuals with CF who harbor F508del-gating and -residual function genotypes [41,42]. Snapwell filters were mounted into Ussing chambers and short circuit currents were measured with a VCC MC6 or VCC MC8 multichannel voltage-current clamp amplifier (Physiologic Instruments, Reno, NY, USA). Apical and basolateral chambers contained a symmetrical chloride solution, and buffers were maintained at 37 °C and continuously circulated by carbogen gas lift. After currents equilibrated, 100 μM Amiloride (Selleckchem, Houston, TX, USA) was added to the apical chamber to inhibit the epithelial sodium channel (ENaC). 10 μM forskolin (Selleckchem, Houston, TX, USA) and 100 μM 3-isobutyl-1-methylxanthine (IBMX, Sigma-Aldrich, Darmstadt, Germany) were added to the basolateral chamber to activate chloride transport through cAMP-mediated channel opening. Finally, ivacaftor was added to the apical chamber to potentiate CFTR activity. After currents plateaued, 10 μM Inh-172 (Selleckchem, Houston, TX, USA) was added to the apical chamber to block CFTR-mediated current. Acquire and Analyze software (Physiologic Instruments, Reno, NV, USA) was used to acquire data, and drop in current (ΔI_sc_) after addition of Inh-172 was used to quantify CFTR-specific function.

### 2.14. Bulk RNA-Sequencing Analysis on the Differentiated ALI Culture of Primary Human NE Cells

To assess effect of readthrough agents and CFTR modulators on differential RNA expression, RNA was extracted from the human NE cells growing on ALI cultures after functional measurement of CFTR activity. Beginning with four fastq files per sample, corresponding to two lanes of paired-end reads, alignment and quantification of RNA-sequencing results was performed using salmon version 1.6.0 and Python version 3.7.12 using a salmon index created using the GENCODE.v39 reference. The quant.sf files produced from these runs were then converted into DESeqDataSet format using tximport version 1.22.0 in R version 4.1.2 and differential expression analysis was performed using DESeq2 version 1.34.0. Plots were generated using ggplot2 version 3.3.6. Expression of *SCNN1B* and *GAPDH* were evaluated before and after treatments.

### 2.15. Statistical Analysis

Statistical analysis was performed using GraphPad Prism (GraphPad Software Inc., San Diego, CA, USA). One-way ANOVA was performed followed by Dunnett’s test for multiple comparisons. *p*-values < 0.05 of were considered significant.

## 3. Results

### 3.1. Nonsense Variants in the 5′ Region Use AUG Codon at Position M265 as an Alternative Downstream Translation Start Site

**Figure 1:**
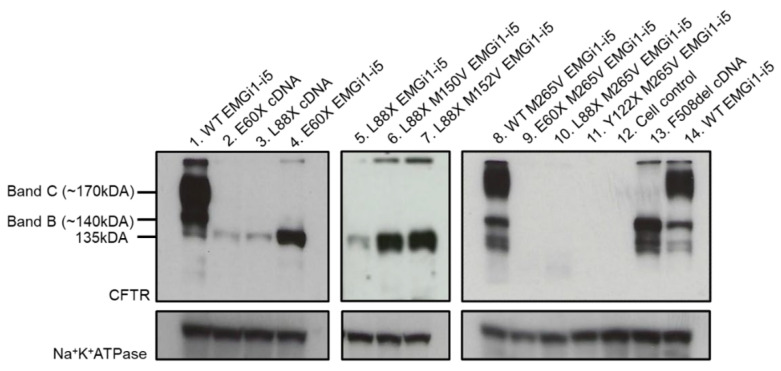
AUG codon at position M265 is used as an alternative downstream translation initiation site for N-terminal *CFTR* nonsense variants. Immunoblot showing steady state levels of CFTR protein. Band B is immature, core-glycosylated CFTR protein and band C is mature, complex-glycosylated CFTR protein. Na^+^K^+^ATPase shown on bottom as a loading control. Protein lysates were collected from HEK293 cells transiently transfected with intronless cDNA or *CFTR*-EMGs bearing N-terminal nonsense variants and downstream methionine mutations. HEK293 cells expressing WT-*CFTR*-EMGi1-i5 and intronless F508del CFTR served as positive controls, and non-transfected HEK293 cells as a negative control (cell control). Immunoblot was probed with anti-CFTR antibody, 596.

Variants that introduce premature termination codons with the 5′ region of *CFTR* may evade NMD through a mechanism of translation initiation at a downstream AUG codon, resulting in synthesis of a N-terminally truncated protein. In *CFTR*, AUG codons at positions M150, M152, and M265 in exons 4 and 7 have all been shown to operate as alternative start sites [34,46]. To determine whether truncated proteins are generated from transcripts bearing N-terminal nonsense variants, we utilized *CFTR* expression minigenes [32,37,47,48,49] that contain full length *CFTR* cDNA and select abridged or full-length introns, and reproduce splicing patterns of native *CFTR*. The inclusion of intron sequences in EMGs is crucial for studying variants that undergo NMD as it allows for formation of exon junction complexes, which are essential for NMD to occur. EMG constructs were generated containing *CFTR* introns 1 through 5, and the variants E60X, L88X, and Y122X were individually introduced via site-directed mutagenesis (SDM). Methionine at positions 150, 152, and 265 were also individually mutated to valine via SDM. EMGs containing E60X, L88X, Y122X and wildtype *CFTR* were individually transiently transfected into Human Embryonic Kidney 293-Flpin (HEK293) cells. Immunoblotting of protein lysates extracted from HEK293 cells showed intronless E60X-*CFTR* cDNA and L88X-*CFTR* cells generate a truncated CFTR product, indicating that this protein is not formed by alternative splicing, but rather through translation initiating at a downstream AUG codon (~135 kDA; Figure 1, lanes 2 and 3). The same size protein is observed in cells transfected with WT-*CFTR*-EMGi1-i5, E60X-*CFTR*-EMGi1-i5 and L88X-*CFTR*-EMGi1-i5 (~135 kDA; Figure 1, lanes 1, 4 and 5) consistent with downstream translation initiation and formation of an N-terminally truncated protein product.

When methionine at position 150 or position 152 are mutated to valine in these cell lines, CFTR protein product remains present (~135 kDA; Figure 1, lanes 6 and 7). However, when methionine at position 265 is mutated to valine, this results in loss of CFTR protein for cells expressing variants E60X, L88X, and Y122X (Figure 1, lanes 9–11). Variant M265V results in no visible changes in protein processing of WT-*CFTR*-EMGi1-i5 (Figure 1, lane 8). This indicates that downstream translation initiation of 5′ *CFTR* nonsense variants is occurring at the AUG codon at position M265. Alternate initiation allows downstream exon-junction complexes to be removed from the transcript, thereby providing a likely explanation for evasion of the NMD pathway by *CFTR* transcripts bearing a nonsense variant 5′ of codon 265.

### 3.2. Variant L88X Is Responsive to Treatment with Readthrough and CFTR Modulators

**Figure 2:**
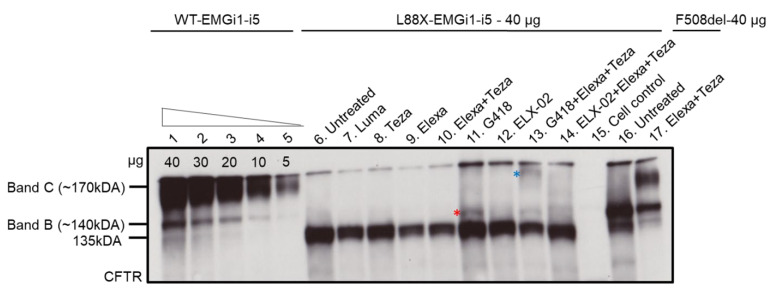
Readthrough and CFTR modulator combination restores full-length, mature, complex-glycosylated CFTR protein for variant L88X. Immunoblot showing steady state levels of immature, core-glycosylated CFTR protein (band B) and mature, complex-glycosylated CFTR protein (band C). Protein lysates were collected from stably transfected HEK293 cells expressing L88X-*CFTR*-EMGi1-i5 to show response to CFTR modulators (lumacaftor 3 µM, tezacaftor 3 µM, and elexacaftor 3 µM), readthrough (G418 200 µM, ELX-02 50 µM), or their combination. All treatments were given for 24 h. µg corresponds to the amount of protein loaded. Triangle indicates decreasing amounts of WT-EMGi1-i5 protein loaded, as indicated by respective labels. Red asterisk indicates full-length core-glycosylated CFTR, and blue asterisk indicates full-length, mature, complex-glycosylated CFTR. HEK293 cells expressing WT-*CFTR*-EMGi1-i5 and intronless F508del CFTR served as positive controls, and non-transfected HEK293 cells as a negative control (cell control). Immunoblot was probed with anti-CFTR antibody, 596.

Immunoblotting of protein lysates extracted from HEK293 cells showed that cells stably transfected with WT-*CFTR*-EMGi1-i5 primarily produce mature, complex-glycosylated CFTR protein (band C) and low levels of immature, core-glycosylated CFTR protein (band B; Figure 2, lanes 1–5). F508del-*CFTR* produced predominately immature protein, as expected from this variant (Figure 2, lane 16). As indicated previously, L88X-*CFTR*-EMGi1-i5 untreated cells generates a CFTR-specific core glycosylated product (~135 kDA; Figure 2, lane 6) consistent with downstream translation initiation at M265 and formation of an N-terminally truncated protein product. Addition of lumacaftor, tezacaftor, or elexacaftor alone, or combination treatment of elexacaftor and tezacaftor did not result in any visible changes in CFTR protein processing in L88X-*CFTR*-EMGi1-i5 cells (Figure 2, lanes 7–10). To explore readthrough potential in the L88X variant, both G418 and ELX-02 readthrough compounds were studied. Addition of G418 resulted in production of a higher molecular weight protein (~140 kDa, red asterisk; Figure 2, lane 11), consistent an alternate amino acid being placed at codon 88 due to readthrough and generation of full-length core-glycoslyated CFTR (compare with band B of F508del; Figure 2, lane 16). Addition of elexcaftor-tezacaftor in combination with G418 resulted in production of a protein of ~170 kDa, consistent with the size of full-length, mature, complex-glycosylated CFTR (blue asterisk; Figure 2, lane 13). The appearance of a new band upon elexcaftor-tezacaftor combination treatment suggests that the folding of the core-glycoslyated CFTR produced by G418 readthrough has been partially corrected (note continued presence of ~140 kDa protein in G418 + elexa + teza lane, Figure 2, lanes 11 and 13). Although difficult to discern, addition of readthrough compound ELX-02 alone, or in combination with elexacaftor-tezacaftor, also resulted in a faint band at ~170 kDA, consistent with mature CFTR (Figure 2, lane 14).

### 3.3. G418 Readthrough and Modulator Treatment of L88X Results in Dose-Dependent Recovery of CFTR Function

**Figure 3:**
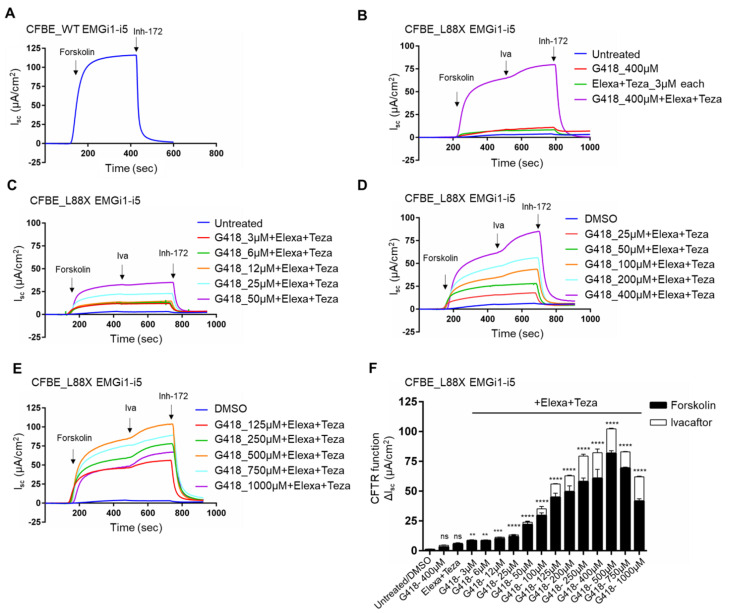
Combination treatment of readthrough G418 with CFTR modulators restores L88X CFTR function in a dose dependent manner in CFBE stable cells. (**A**) A representative short circuit current (Isc) assay in CFBE cells stably expressing WT-*CFTR*-EMGi1-5 grown on snapwells. CFBE cells were mounted on Ussing chambers to measure CFTR mediated chloride activity as a proxy to CFTR function. Forskolin (10 µM) was added to the basolateral chamber, followed by CFTR inhibitor Inh-172 (10 µM) added to the apical chamber. Average CFTR function for all WT-*CFTR*-EMGi1-5 CFBE stable cells was 123 ± 11 µA/cm^2^. (**B**–**E**) Isc assay of CFTR channel function in CFBE cells stably expressing L88X-*CFTR*-EMGi1-i5. Cells were incubated for 24 h with varying concentrations of readthrough compound G418 (3 µM–1000 µM), CFTR correctors elexacaftor (3 µM) and tezacaftor (3 µM), or both. Forskolin (10 µM) was added to the basolateral chamber, followed by potentiator ivacaftor (10 µM), and CFTR inhibitor Inh-172 (10 µM) added to the apical chamber. (**F**) Stacked bar plots indicate effect of G418 readthrough concentration and modulator treatment on L88X-*CFTR*-EMGi1-i5 CFBE cells. Change in Isc (ΔIsc) was defined as the current inhibited by Inh-172 after sustained Isc responses achieved upon stimulation with forskolin alone or sequentially with ivacaftor. ** indicates *p ≤* 0.01, *** indicates *p* ≤ 0.001, **** indicates *p* ≤ 0.0001.

L88X-*CFTR*-EMGi1-i5 was stably integrated into a single genomic site in CF bronchial epithelial (CFBE) Flpin cells. CFBE cells were grown on filters to allow for polarization, and filters were then mounted into Ussing chambers. Addition of forskolin stimulated production of cAMP, resulting in activation of CFTR channels. This was followed by addition of CFTR inhibitor, Inh-172, to block CFTR-mediated current, and CFTR-specific current was measured (ΔIsc ± SEM). WT CFTR was first assessed by measuring current in CFBE cells stably expressing WT-*CFTR*-EMGi1-i5. The average function for untreated WT-*CFTR*-EMGi1-i5 was 123 ± 11 µA/cm^2^ (Figure 3A). In CFBE cells expressing L88X-*CFTR*-EMGi1-i5, very low levels of chloride current were generated (0.51 ± 0.1 μA/cm^2^), equivalent to less than 1% of WT function, which is consistent with the disease severity of the L88X variant (Figure 3B). CFTR currents were unchanged after addition of readthrough compound G418 (400 μM), a combination of elexacaftor and tezacaftor (3 μM each), or upon acute treatment with ivacaftor (Trikafta). However, addition of Trikafta in combination with G418 (400 μM) resulted in robust recovery of CFTR function, corresponding to approximately 66% of WT levels (82 ± 0.4 μA/cm^2^) (Figure 3B). To determine the responsiveness of L88X-*CFTR* to G418 and Trikafta combination treatment, a dose response study was performed (3 μM–1000 μM). Concentrations of G418 below 12 μM show minimal functional recovery of CFTR (Figure 3C). However, at concentrations of G418 above 25 μM, there is a progressive increase in CFTR current. From 25 μM–400 μM G418, there is continued increase in CFTR response, occurring in a dose-dependent manner (Figure 3D). To explore maximal CFTR recovery from G418 treatment, higher concentrations of G418 (500–1000 μM) in combination with Trikafta were also tested (Figure 3E). There was a continued dose-dependent response in CFTR current, with a drop off in CFTR response at concentrations of 750 μM and above. The highest level of functional recovery observed occurred at a concentration of 500 μM G418, with function corresponding to approximately 83% of WT-CFTR function (102 ± 1.5 μA/cm^2^) (Figure 3F).

### 3.4. ELX-02 Readthrough and Modulator Treatment of L88X Results in Dose-Dependent Recovery of CFTR Function

**Figure 4:**
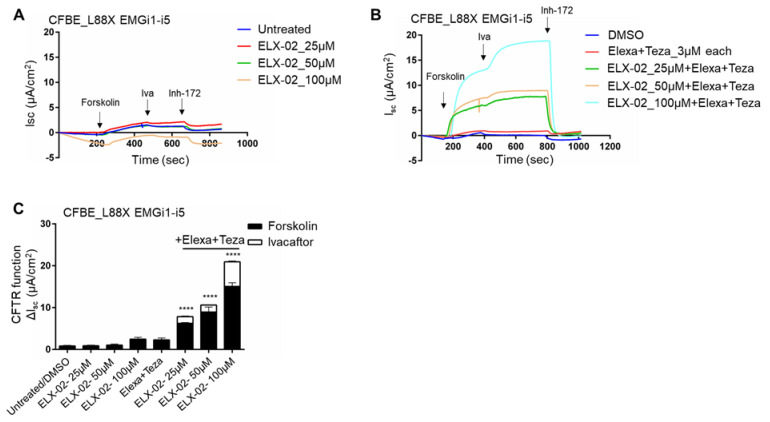
Combination treatment of readthrough ELX-02 with CFTR modulators restores L88X CFTR function in a dose dependent manner in CFBE stable cells. (**A**) Short circuit current (Isc) assay in CFBE cells stably expressing L88X-*CFTR*-EMGi1-5 grown on snapwells. CFBE cells were mounted on Ussing chambers to measure CFTR mediated chloride activity as a proxy to CFTR function. Cells were incubated for 24 h with varying concentrations of readthrough compound ELX-02 (25–100 µM). Forskolin (10 µM) was added to the basolateral chamber, followed by potentiator ivacaftor (10 µM) and CFTR inhibitor Inh-172 (10 µM) added to the apical chamber. (**B**) Isc assay of CFTR channel function in CFBE cells stably expressing L88X-*CFTR*-EMGi1-i5. Cells were incubated for 24 h with varying concentrations of readthrough compound ELX-02 (25–100 µM) and CFTR correctors elexacaftor (3 µM) and tezacaftor (3 µM). Forskolin (10 µM) was added to the basolateral chamber, followed by potentiator ivacaftor (10 µM), and CFTR inhibitor Inh-172 (10 µM) added to the apical chamber. (**C**) Stacked bar plots indicate effect of ELX-02 readthrough concentration and modulator treatment on L88X-*CFTR*-EMGi1-i5 CFBE cells. Change in Isc (ΔIsc) was defined as the current inhibited by Inh-172 after sustained Isc responses achieved upon stimulation with forskolin alone or sequentially with ivacaftor. **** indicates *p* ≤ 0.0001.

Given the known toxicity of G418, there remains the limitation that the concentration necessary to observe substantial CFTR function recovery may be above a reasonable threshold for use in individuals with CF. This limitation highlights the importance of testing additional readthrough therapeutic candidates. Since immunoblotting for the detection of protein is relatively insensitive compared to functional testing, and response to readthrough compounds can differ by cell type (i.e., HEK293 vs. CFBE), we elected to determine whether ELX-02 could enable sufficient readthrough to generate CFTR currents in CFBE cells. Cells bearing L88X-*CFTR*-EMGi1-i5 were treated with ELX-02. No functional recovery of CFTR was observed after ELX-02 treatment with concentrations ranging from 25 μM–100 μM (Figure 4A). However, upon treating these cells with these same concentrations of ELX-02 in combination with Trikafta, there is a robust recovery of CFTR function (Figure 4B). This functional response was dose dependent, with the maximal CFTR recovery occurring at 100 μM ELX-02, corresponding to ~17% of WT function (Figure 4C). These results indicate that readthrough compounds such as ELX-02 will require CFTR modulators to recover therapeutic levels of CFTR function.

### 3.5. RNA Analysis Confirms Presence of L88X Transcript in Nasal Epithelial Cells of Carrier Parent

**Figure 5:**
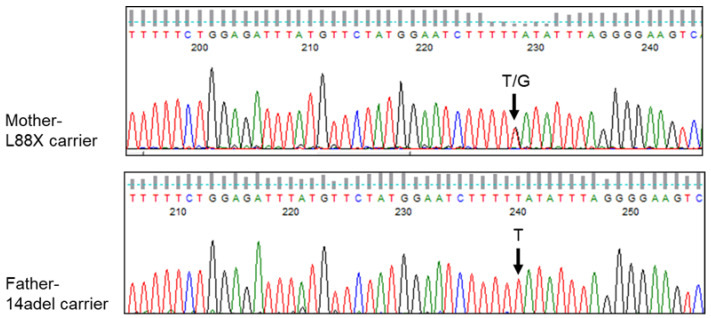
Primary nasal epithelial cells of heterozygous carrier of L88X generate detectable L88X-*CFTR* mRNA. Top panel. Sanger sequencing of RT-PCR of region surrounding L88X. Total RNA was extracted from conditionally reprogrammed primary human nasal epithelial (NE) cells from mother of the proband, who is a carrier for L88X. RT-PCRs were performed using *CFTR*-specific primers to amplify the L88X region. Bottom panel. Sanger sequencing of RT-PCR of region surrounding L88X. Total RNA was extracted from conditionally reprogrammed primary human nasal epithelial (NE) cells from the father of the proband, who is a carrier for 14adel. RT-PCRs were performed using *CFTR*-specific primers to amplify the L88X region.

To further evaluate results in our EMG cell-based system, studies were performed on primary human nasal epithelial (NE) cells obtained from an individual carrying L88X/14adel, and their heterozygous parents. Reverse transcription polymerase chain reaction (RT-PCR) was performed on cDNA from both parents. Sanger sequencing of these products revealed that the mother, who carries L88X, has detectable L88X-*CFTR* mRNA, indicating that this transcript is evading NMD (Figure 5).

### 3.6. Readthrough and Modulator Treatment Restores CFTR Function in Primary Human Nasal Epithelial Cells Bearing L88X

**Figure 6:**
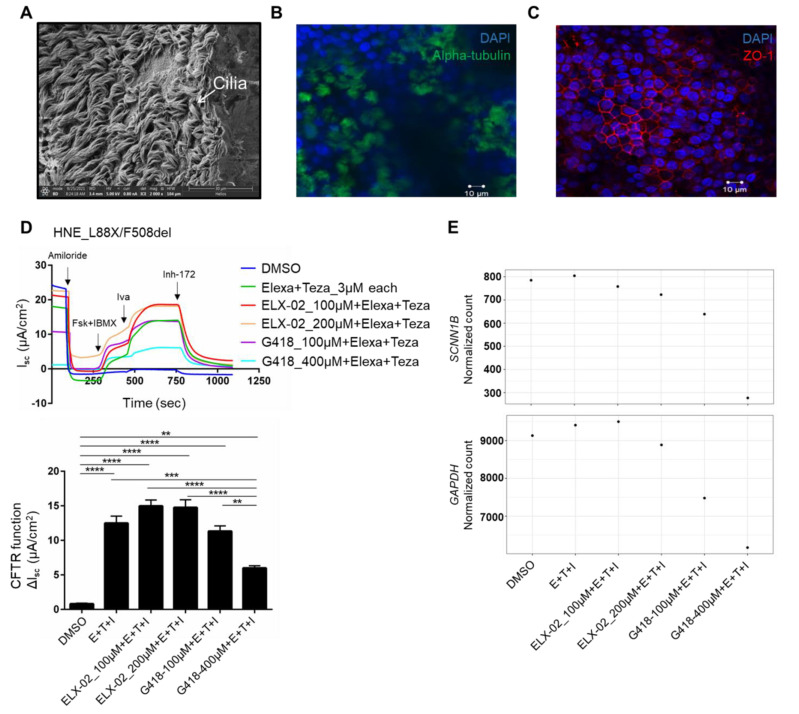
Combination treatment of readthrough G418 or ELX-02 with CFTR modulators restores L88X CFTR function in primary human nasal epithelial cells harboring L88X. (**A**) Scanning electron microscopy of primary human nasal epithelial (NE) cells at air-liquid interface (ALI) culture. NE cells were conditionally reprogrammed using Rho-kinase inhibitor. NE cells were transferred on snapwellls and exposed to air on the apical side to establish ALI culture. (**B**) Confocal microscopy of NE cells following staining of alpha tubulin, a marker of cilia. (**C**) Confocal microscopy of NE cells following staining of ZO-1, a marker of tight junctions. (**D**) Short circuit current (Isc) assay in L88X/F508del NE cells. NE cells were mounted on Ussing chambers to measure CFTR mediated chloride activity as a proxy to CFTR function. Cells were incubated for 24 h with varying concentrations of readthrough compound ELX-02 (100 µM and 200 µM) or G418 (100 µM and 400 µM) and CFTR correctors elexacaftor (3 µM) and tezacaftor (3 µM). Forskolin (10 µM) was added to the basolateral chamber, followed by potentiator ivacaftor (10 µM) and CFTR inhibitor Inh-172 (10 µM) added to the apical chamber. Stacked bar plots indicate effect of ELX-02 or G418 readthrough concentration and modulator treatment on L88X/F508del NE cells. Change in Isc (ΔIsc) was defined as the current inhibited by Inh-172 after sustained Isc responses achieved upon stimulation with forskolin alone or sequentially with ivacaftor. ** indicates *p* ≤ 0.01, *** indicates *p* ≤ 0.001, **** indicates *p* ≤ 0.0001. (**E**) Bulk RNA-sequencing of L88X/F508del NE cells. Total RNA was extracted from the filters after CFTR functional testing. Each point represents one biological replicate. Top panel shows normalized count of *SCNN1B* and bottom panel shows normalized count of *GAPDH* after treatment with ELX-02 (100 µM and 200 µM) or G418 (100 µM and 400 µM) and CFTR correctors elexacaftor (3 µM), tezacaftor (3 µM), and ivacaftor (10 µM).

Human NE cells were conditionally reprogrammed and grown at air liquid interface (ALI). Scanning electron microscopy demonstrates presence of abundant cilia following NE cell differentiation on ALI culture (Figure 6A). Additionally, confocal microscopy with staining of alpha tubulin and ZO-1 as markers of cilia and tight junctions, respectively, also demonstrates well differentiated ALI cultures (Figure 6B,C).

Well-differentiated cells were mounted into Ussing chamber and Amiloride was added to the apical chamber to inhibit the epithelial sodium channel (ENaC). Subsequent addition of forskolin and IBMX activated chloride transport through cAMP-mediated channel opening, and Inh-172 was added to block CFTR-mediated current. CFTR function observed in WT NE cells was 14.4 ± 1.4 μA/cm^2^ (n = 17, 3–11 observations each sample). In L88X/F508del NE cells, untreated/DMSO treated cells generated minimal CFTR function (ΔI_sc_ = 0.80 ± 0.1 µA/cm^2^) corresponding to ~5.5% of WT CFTR function, consistent with the disease severity of these variants. Treatment with Trikafta alone restored significant CFTR function (ΔI_sc_ = 12.49 ± 1.0 μA/cm^2^) corresponding to ~86% of WT, attributable to recovery of F508del CFTR (Figure 6D and bar graph). Addition of ELX-02 to Trikafta treatment of NE cells at 100 µM and 200 µM resulted in further improvement of CFTR function (ΔI_sc_ = 14.9 ± 0.8 μA/cm^2^ and 14.8 ± 1.1 μA/cm^2^) corresponding to ~104% and ~103% of WT CFTR function, respectively (Figure 6D and bar graph). Interestingly, addition of G418 at both 100 µM and 400 µM to Trikafta treatment of NE cells reduced CFTR function (ΔI_sc_ = 11.3 ± 0.7 μA/cm^2^ and 6.0 ± 0.3 μA/cm^2^), corresponding to ~78% and ~42% of WT, respectively, (Figure 6D and bar graph). Furthermore, G418 resulted in reduced amiloride-sensitive β-ENaC inhibition at 100 µM and near-zero inhibition at 400 µM (Figure 6D).

To determine if this reduction in amiloride response observed upon treatment with G418 was a physiological effect on β-ENaC activity or a transcriptomic effect, we evaluated *SCNN1B*, which encodes for β-ENaC, by bulk RNA-sequencing. Analysis revealed that addition of G418 resulted in marked reduction of not only *SCNN1B* mRNA expression, but also reduction of housekeeping gene *GAPDH* expression (Figure 6E), indicating its toxicity to primary NE cells at both 100 µM and 400 µM. Interestingly, ELX-02 in combination with CFTR modulators did not show any marked differences in the mRNA expression of both *SCNN1B* and *GAPDH* (Figure 6E), indicating ELX-02 is non-toxic to NE cells.

Overall, these observations in primary human NE cell studies indicate that 5′ nonsense variants that escape NMD may be treatable though a combination approach of readthrough and CFTR modulator therapy.

## 4. Discussion

PTC-generating variants remain difficult to therapeutically target due to the absence of targetable protein. While readthrough therapeutics have been developed to overcome this challenge, stable mRNA must be present on which for readthrough molecules to act. NMD hinders readthrough molecules ability to produce full-length transcript, as the PTC-bearing transcript is subject to decay. It has previously been shown that *CFTR* transcripts bearing nonsense variants in the 5′ region do not undergo NMD [32], likely through a mechanism where translation begins at one of several downstream AUG codons. Here, we investigate readthrough potential in N-terminal *CFTR* variants E60X, L88X, and Y122X. Analysis of downstream AUG codons revealed that mutation of methionine at position 265 results in loss of CFTR protein, evidence that M265 is a site of alternative translation initiation for these variants. However, we show that the N-terminally truncated CFTR generated by translation initiation at AUG at M265 does not respond to CFTR modulators. Evaluation of L88X in CFBE stable cell line reveals that readthrough compounds can be used to restore functional CFTR protein, but requires addition of CFTR protein modulators. L88X transcript is comparable to WT transcript levels in primary NE cells, indicating that this variant is escaping NMD. Recovery of CFTR function occurs upon addition of ELX-02 readthrough and CFTR modulators in NE cells indicating that treatment of N-terminal *CFTR* variants may be possible through this therapeutic approach.

As previously demonstrated [32] and as we exhibited here, N-terminal variants undergo downstream translation initiation, resulting in evasion of NMD. Recently, evidence suggests that nonsense *CFTR* variants G542X, R1162X, and W1282X require the NMD factors UPF2 and UPF3 for NMD to occur, but the N-terminal variant Y122X is not recognized by these factors [50]. Additionally, mRNA levels of Y122X-*CFTR* are higher compared to mRNA levels of *CFTR* bearing other nonsense variants [50]. This could be explained by the mechanism in which downstream translation initiation causes these NMD factors to be removed from the transcript, resulting in evasion of NMD. Studies investigating additional N-terminal *CFTR* variants, including L88X, and what NMD factors recognize these variants, will strengthen evidence of by which mechanism these variants are evading NMD.

Translation initiation is a stochastic event that depends on the strength of the surrounding context of the start codon [51,52]. Start codons are recognized at varying efficiencies depending on similarity to the Kozak consensus sequence, GCCRCCAUGG (R = A or G) [52]. More recently, the strongest translation initiation sequence has been expanded to the motif RYMRMVAUGGC (Y = C or U, M = A or C, V = G, C or A) [53]. The −3 and +4 positions (with A in AUG as +1) of the consensus sequence are known to have a particularly strong effect [52,54]. These considerations will influence how efficiently downstream AUG codons in *CFTR* are used for alternate translation initiation. AUG codons at positions M150 and M265 both contain the conserved purine at the −3 position, where position 152 contains a pyrimidine, making efficiency of M152 more sensitive to changes in surrounding positions [52]. None of the three AUG codons studied here have the conserved G at the +4 positions, likely reducing their translation initiation efficiency [54]. However, there are many AUGs that serve as start codons even though their sequence context is considered to be low efficiency [51,52]. Between AUG codons at positions M150, M152, and M265, the AUG at M265 is most similar to the consensus sequence, potentially impacting preference of this AUG over others as the site of downstream translation initiation, as observed here.

Assessing variants that introduce PTCs requires model systems that accurately represent mRNA levels in vivo. Primary cells are difficult to procure, especially for rare variants, which are often found in *trans* with variants that produce stable mRNA and/or protein. Cell lines expressing cDNA constructs are not sufficient to study PTC-generating variants, as the absence of introns prevents NMD from occurring. Prior studies have shown that our expression minigene system is a reliable model for studying PTC-generating variants [32,37,48,49,55,56]. In this study, HEK293 cells expressing EMG constructs provide the advantage of rapid analysis of mRNA levels and protein processing [57]. Through this EMG system, we show that untreated cells bearing N-terminal variants E60X or L88X both produce product indicative of N-terminally truncated CFTR protein. Loss of CFTR protein upon mutation of methionine at 265 signifies that translation initiation of 5′ nonsense variants E60X, L88X, and Y122X is occurring at M265. Although this N-terminally truncated protein cannot be activated by CFTR modulators, this mode of alterative translation initiation would remove downstream EJCs that are crucial in triggering NMD [58]. Indeed, presence of the truncated protein indicates that mRNA in this cell-based system must be present and NMD has been averted. Prior to position 265, there are 21 naturally occurring N-terminal nonsense variants in *CFTR*, with a total allele count of 783 (https://CFTR2.org; accessed on 30 August 2022), which could potentially evade NMD through this mechanism. Together, these results are consistent with the concept that translation initiation at AUG at M265 allows for removal of downstream EJCs, resulting in evasion of NMD and formation of the N-terminally truncated CFTR protein. Our study does have the limitation that we have only observed downstream initiation through immunoblotting, and further evaluation through mass spectrometry and ribosome profiling could more precisely determine if translation initiation is indeed occurring at AUG at position M265 [51,59,60]. Mass spectrometry could also determine if ribosomes are scanning past all AUGs prior to initiation at AUG at M265, or if there is another mechanism by which ribosomes are reaching this position, such as through multiple mRNA isoforms with alternative transcription start sites.

Variants that naturally evade NMD are ideal targets for readthrough compounds due to the stability of the mRNA. Likelihood of restoration of CFTR function may be dependent on what amino acid is being inserted at the site of the PTC, since the amino acid inserted by readthrough is often different than the amino acid present in the wildtype protein. Studies investigating amino acid insertion have found that stop codon identity and local mRNA sequence context play an important role in determining what amino acid is inserted at a PTC [17,61,62]. Additional evaluation into identity of the amino acid that is inserted will aid in predicting likelihood of protein function restoration. Moreover, effectiveness of PTC suppression by readthrough compounds has been shown to be influenced by surrounding sequence context of the nonsense variant, including both the identity of the stop codon the PTC generates, and the nucleotides flanking the variant [16,22,62,63,64]. Nonsense mutations can be found anywhere within the coding sequence, and thus sequence context will ultimately be highly variable between different variants targeted for treatment. Additional considerations when evaluating efficacy of readthrough compounds for therapeutic efforts include normal termination codon (NTC) readthrough. NTC readthrough remains a limiting factor when considering readthrough therapies from a treatment perspective. However, it has been postulated that readthrough compounds may be more likely to readthrough PTCs than NTCs due to evolutionary pressure for NTCs to remain high effective [16]. Supporting this prediction, researchers have discovered through ribosome profiling that stop codons within 3′UTRs are more likely to be subject to readthrough than NTCs [16]. Additionally, different readthrough molecules may have greater preference for readthrough of PTCs over NTCs. Preliminary data suggests that treatment with readthrough compound ELX-02 results in significant PTC readthrough, but does not result in aberrant protein products indicative of NTC readthrough [65]. Notably, ELX-02 has been shown to have reduced toxicity compared to G418, which is the most commonly used molecule for readthrough of PTCs [65,66,67]. This known toxicity has been a major limitation for the use of aminoglycoside antibiotics in a clinical treatment setting for PTC readthrough [11,30,31,68,69].

For some N-terminal variants, addition of CFTR modulators alone may be sufficient to assist in protein processing and generate truncated yet mature CFTR. However, mutations beyond a certain point may not be recoverable given the binding site of CFTR correctors. Tezacaftor and lumacaftor are known to bind to CFTR in the first transmembrane spanning domain, and mutation of residues in this region can significantly impact efficacy of these correctors [70]. Addition of readthrough compounds could alleviate this issue, but identity of the amino acid that is inserted at the PTC can also greatly impact CFTR folding. In HEK293 cells, addition of elexacaftor-tezacaftor in combination with G418 restores product of full-length, mature CFTR protein, indicating that modulators are able to correct the folding defect introduced by readthrough alone. Interestingly, addition of ELX-02 and modulator therapy produces this same mature CFTR protein product, but the product is difficult to discern. The readthrough effect observed with ELX-02 is likely at or below the limits of detection for immunoblotting. Significant recovery of CFTR function is observed upon treatment with ELX-02 and modulators in L88X CFBE cells, although not as robust with G418. However, the concentration necessary to reach significant clinical benefit may exceed the tolerable threshold given the toxicity of G418 [71]. Given the tolerability of ELX-02, higher doses may be possible as a treatment option and may achieve greater functional response. These results are consistent with previous studies regarding successful CFTR response to readthrough therapy [72]. Notably, high throughput screens have identified new small molecule compounds that may induce readthrough, which can be tested in combination with modulator therapy as an alternate approach to restoring CFTR function [73]. Additionally, anticodon engineered transfer RNAs (ACE-tRNAs) should be considered for further investigation given their ability to suppress PTCs and encode for a cognate amino acid, reducing the issue of protein misfolding and trafficking that can occur with readthrough compounds [74,75]. Taken together, these results indicate that readthrough therapy will require addition of CFTR modulators to recover CFTR function to therapeutic levels.

We additionally demonstrate efficacy of readthrough and modulator treatment through CFTR functional analysis in primary NE cells bearing L88X-*CFTR*. L88X mRNA is detectable in primary cells, which indicates that L88X is efficiently evading NMD in the native *CFTR* context. We find that in L88X/F508del NE cells, treatment with G418 and Trikafta does not significantly improve CFTR function. However, our study does have the limitation that addition of Trikafta alone significantly restores function for F508del-CFTR, making it difficult to observe minor additional functional improvements. Further studies in an individual homozygous for L88X may aid in improving our understanding of G418 utility.

Overall through this study we demonstrate that mRNA stability is a critical consideration that has been previously overlooked when determining the utility of readthrough therapeutics. In a variant that naturally evades NMD, L88X, CFTR function can be dramatically restored through readthrough treatment when combined with CFTR modulators. Additional N-terminal variants that may evade NMD should be prioritized when evaluating the therapeutic potential for readthrough compounds in a clinical setting, especially ELX-02, which is already in clinical trials. Additionally, N-terminal variants in other diseases should be thoroughly assessed for presence of stable mRNA and considered for evaluation of therapeutic benefit of readthrough.
